# Annotations

**Published:** 1893-02-04

**Authors:** 


### ANNOTATIONS.

**A Masseuse's "Secret."**

"AN ENQUIRER" asks us to insert the following in our "Queries" column for a reply: "How can a remedy be put to the test in England, which was tried innumerable times in India very successfully, as a positive preventive to (*sic*) hydrophobia without my having to give up the secret?" As a matter of practical business, the question is ridiculous: as a matter of morals, it is disgraceful. The only way to test a supposed preventive of hydrophobia would be to administer the drug to a large number of persons—say half-a-million—and then to cause them all to be well bitten by mad dogs. There would, if not one of the bitten persons took the disease, be established a presumption that the article was a prophylactic. But what sane person would make such an experiment upon himself, and more especially with a "secret" remedy? Of course, it is open to make the initial experiments upon half-a-million dogs; but such experiments would by no means be conclusive for human beings. We do not, however, notice the question for the purpose of showing how the experiments might be made, but for the purpose of trying once more to make the public see the enormity of keeping a remedy secret which might possibly have the power of saving life. We protest, as we have often done before, that the maxims of ordinary business do not apply to the life-saving art of medicine. If any man had a "secret" remedy, which was a real "remedy" and cure for, say, cancer, consumption, or hydrophobia, and kept that remedy secret for the mere purpose of making money by it, he would be a monster and not a man, and would deserve to be hanged as a murderer. For would he not be a real murderer, and that on an unparalleled scale? Would he not be keeping locked up in his own bosom a secret which, if revealed, would save tens of thousands of lives? It is impossible for us even to understand why the public should have any confidence whatever in a man who professes to have a cure for a mortal disease and yet keeps it a secret! Why, the man stands self-confessed a prodigy of baseness! What would anybody think if a fireman in a burning house were to begin to make money terms with the terrified inmates before he would consent to carry them outside the blazing pile! But the fireman's wickedness would be as nothing compared with that of the man in possession of a remedy for every case of a certain kind of mortal disease, who should keep it secret for his own profit. As for the "masseuse" who sends us the question, if she is not ashamed of herself, her conscience must be in a very embryonic condition. All dealers in "secret" remedies ought to be sent to the treadmill.

**Jack Tar's Cookery.**

"Is it impossible that Britannia, who confessedly rules the waves, should attend to the victuals a little, and that meat should be well cooked under a Union Jack?" This question was originally put by Thackeray, but by how many has it been echoed since? It has recently been the subject of inquiry by a representative committee, and the verdict is that though shipowners as a rule provide good food, the cooks are still sent by the proverbial authority. We do not now speak so much of the great passenger steamers, with their trained and seasoned cooks, who will make you an omelette in a tornado and a *vol-au-vent* in the midst of a hurricane. But what of Jack Tar in the forecastle of a sailing ship on a long voyage? His diet cannot be greatly varied in kind; it must be salted or tinned meat of some sort, or at least something that will keep for an indefinite time. It is fortunate that the average sailor has the digestion of an ostrich, or he would be tempted to grumble at his fare more often than he does. As it is, complaints, well or ill founded, are often enough heard. But, as often as not, the fault lies in the cookery. The food, such as it is, is not made the most of—the most savoury, the most digestible. To improve this state of affairs, schools of nautical cookery have been established in Glasgow, where young sailors don the cook's white apron and learn to make appetising Irish stews and sea pies with such materials at their command as they might hope to have on board ship. They are skilled in "plum-duff"—an idealised "duff" which even a landsman need not despise—and use the rolling-pin like practised hands. The committee which has just concluded its deliberations have recommended the establishment of such schools in every port. There can be no doubt that this would have an important influence on the food, and consequently on the health of seamen. The value of food is minimised by bad cooking, and in a ship where every pound of extra weight is an additional burden it is common sense to insist on everything being put to the utmost use. Shipowners are not unwilling to give good food; from their point of view a ship delayed by the bad working of a sickly crew costs more than the difference between good food and bad. Sometimes, indeed, they are cheated by dishonest contractors and officials; but when they put themselves to the test of eating the food left at the end of a voyage and pronounce it very good, one is forced to conclude that it is the cooking that gives cause for complaint. If schools of nautical cookery improve this, shipowners as well as seamen will be the gainers by them.

**Hospital Saturday in London.**

WE have great pleasure in being able to report that the expenses of the Hospital Saturday Fund for 1892 show a reduction of about £700 as compared with the previous year, and that the working expenses have been so reduced of late that they now amount to very little more than 10 per cent. of the receipts. Mr. Reginald B. Acland, the Chairman of the Council, has rendered yeoman service, and his whole-hearted devotion to the best interests of this movement entitle him to public thanks. The Hospital Saturday Fund is handicapped by the ticket system, and its managers have, we understand, come to the conclusion that it would be better if the ticket system could be entirely abrogated so far as their workshops are concerned. The hospitals feel, on the one hand, that the demands made by the Hospital Saturday Fund on their resources are out of proportion to the amount of grants received from the Fund; the artisans, on the other hand, are impressed with the feeling that they are entitled to hospital relief for themselves and their families if they subscribe, according to their means, to the Saturday Fund. We advocate the institution of letters of introduction, signed by an accredited representative of each workshop, which would entitle the bearer to examination by a member of the hospital staff, and to such treatment as the medical or surgical necessities of each case may warrant. At the end of each year the hospitals should sort these letters of recommendation, and make out an account for each workshop, showing the amount and value of the relief given to the employés who have used the hospital. Detailed and total lists of these results should be sent in to the Council of the Hospital Saturday Fund, as well as to each workshop. They would show, in the majority of cases, that the amount of relief given by the hospitals was much greater in value than the contributions received. This would put the artisans of London upon their mettle, and we believe that the total contributions on Hospital Saturday in London would speedily be increased to at least £50,000. Our plan has the further advantage that it would abolish for ever the unsatisfactory system which permits the Central Council to receive money from all the workshops of London, and to distribute the patronage resulting from it, instead of leaving each workshop to secure for itself, by virtue of its own contributions, any such patronage and such medical aid as the employés may require.

